# Guanine Deaminase in Human Epidermal Keratinocytes Contributes to Skin Pigmentation

**DOI:** 10.3390/molecules25112637

**Published:** 2020-06-05

**Authors:** Joon Min Jung, Tai Kyung Noh, Soo Youn Jo, Su Yeon Kim, Youngsup Song, Young-Hoon Kim, Sung Eun Chang

**Affiliations:** 1 Department of Dermatology, Asan Medical Center, University of Ulsan College of Medicine, 88 Olympic-ro 43-gil, Songpa-gu, Seoul 05505, Korea; bban29@hanmail.net (J.M.J.); premed@naver.com (T.K.N.); sooyounyda@gmail.com (S.Y.J.); u2u2star@naver.com (S.Y.K.); 2 Department of Biomedical Sciences, Asan Medical Center, University of Ulsan College of Medicine, 88 Olympic-ro 43-gil, Songpa-gu, Seoul 05505, Korea; ysong@amc.seoul.kr; 3 Department of Pharmacology, Asan Medical Center, University of Ulsan College of Medicine, 88 Olympic-ro 43-gil, Songpa-gu, Seoul 05505, Korea

**Keywords:** guanine deaminase, melanogenesis, UV radiation

## Abstract

Epidermal keratinocytes are considered as the most important neighboring cells that modify melanogenesis. Our previous study used microarray to show that guanine deaminase (GDA) gene expression is highly increased in melasma lesions. Hence, we investigated the role of GDA in skin pigmentation. We examined GDA expression in post-inflammatory hyperpigmentation (PIH) lesions, diagnosed as Riehl’s melanosis. We further investigated the possible role of keratinocyte-derived GDA in melanogenesis by quantitative PCR, immunofluorescence staining, small interfering RNA-based GDA knockdown, and adenovirus-mediated GDA overexpression. We found higher GDA positivity in the hyperpigmentary lesional epidermis than in the perilesional epidermis. Both UVB irradiation and stem cell factor (SCF) plus endothelin-1 (ET-1) were used, which are well-known melanogenic stimuli upregulating GDA expression in both keratinocyte culture alone and keratinocyte and melanocyte coculture. GDA knockdown downregulated melanin content, while GDA overexpression promoted melanogenesis in the coculture. When melanocytes were treated with UVB-exposed keratinocyte-conditioned media, the melanin content was increased. Also, GDA knockdown lowered SCF and ET-1 expression levels in keratinocytes. GDA in epidermal keratinocytes may promote melanogenesis by upregulating SCF and ET-1, suggesting its role in skin hyperpigmentary disorders.

## 1. Introduction

Skin hyperpigmentation is caused by the interplay between melanocytes and neighboring cells of keratinocytes (KCs), fibroblasts, endothelial cells, and inflammatory cells [1,2,3,4,5,6]. Among them, KCs may have the most important role as they produce abundant melanogenic mediators. KCs, the major type of skin cells, act as the first barrier to UV or environmental stress. KC production and/or growth factor and cytokine secretion are essential stress response mechanisms in response to UV irradiation, inflammatory signals, or skin injury [1,2,3]. Indeed, many paracrine factors secreted from epidermal KCs upon stimulation regulate melanogenesis. Alpha-melanocyte-stimulating hormone (α-MSH) and adrenocorticotropic hormone (ACTH) are key melanogenic stimulators secreted by human epidermal KCs upon UV irradiation [7]. When bound to the melanocortin 1 receptor expressed on melanocytes, they activate the cAMP-protein kinase A/cAMP response element-binding protein pathway [8], which results in upregulation of microphthalmia-associated transcription factor (MITF) [9]. MITF increases the expression level of tyrosinase, the rate-limiting enzyme in melanin biosynthesis, which promotes melanogenesis [10]. Studies by Imokawa et al. [2,3,11,12] focused on alterations in the cytokine–paracrine network known to regulate pigmentation, and demonstrated that endothelin-1 (ET-1) from KCs is mainly responsible for skin hyperpigmentation in senile lentigo [3]. ET-1 binds to endothelin receptors on melanocytes and regulates melanin production [2]. The expression of endothelin receptors is highly elevated by UV irradiation with subsequent enhancement of the ET-1/endothelin receptor type B pathway, which plays an important role in UVB-induced melanogenesis by regulating MITF phosphorylation in normal human melanocytes (NHMs) [12]. Stem cell factor (SCF) and its receptor KIT contribute to melanogenesis [3]. In response to UV exposure, the levels of both SCF and membrane-bound KIT are increased in human epidermal KCs and NHMs, respectively, which results in enhanced melanin production [13]. SCF from KCs is overexpressed in the epidermis of senile lentigo patients, but the role of the secretory form of SCF is unclear [11].

The search for other unknown melanogenic factors produced by epidermal KCs is ongoing. Melasma is a UV irradiation and/or inflammation-aggravated hyperpigmentary disorder [14]. Similar to melasma, Riehl’s melanosis (RM) is characterized by diffuse hyperpigmentary patches on the face and neck and is aggravated by UV irradiation and/or inflammation [15,16]. In our previous study, we found that guanine deaminase (GDA) mRNA expression was 5 to 14-fold higher in melasma lesion tissue than in non-lesion tissue [17]. In purine metabolism, guanine is converted into xanthine by GDA, a ubiquitous aminohydrolase enzyme. Xanthine oxidase further metabolizes xanthine into uric acid, producing reactive oxygen species which can be involved in GDA-induced cellular senescence in human KCs [18]. GDA shows tissue-specific expression in higher eukaryotes [19,20]. Previous histochemical studies have reported that GDA is present within the cytoplasm of liver, kidney, small intestine, and central nervous system cells, but not in fibrous tissue and inflammatory cells [21,22,23,24,25]. As GDA is ubiquitous and functions as a purinergic system in human cells, it may be involved in diverse signaling pathways. GDA can regulate dendritic arborization of neurons and neuronal development [26,27,28]. The embryonic origin of melanocytes is neural crest cells [29]. A few studies associate GDA with skin disorders. Kizaki et al. have reported that GDA enzyme activity was higher in the epidermis of the patients with psoriasis than in the normal epidermis [30]. A recent study suggested GDA, mainly expressed in KCs in human skin, is involved in UV-induced senescence in seborrheic keratosis lesions by producing guanine metabolites [18]. The HPRT1 gene encodes hypoxanthine-guanine phosphoribosyltransferase, which is involved in the purine salvage pathway by catalyzing the conversion of hypoxanthine to inosine monophosphate and guanine to guanosine monophosphate [31]. Although there has been no research on the relationship between GDA and skin pigmentation, Lesch-Nyhan disease (in which HPRT1 gene mutation with subsequent alterations in purine metabolism exist) presents with reticulated skin dyspigmentation and may suggest the role of GDA in skin pigmentation [31,32]. Therefore, in this study, we analyzed the possible involvement of KC-derived GDA in hyperpigmentation.

## 2. Results

### 2.1. Hyperpigmented Skin Lesions of RM are Associated with Increased GDA Expression

This study was approved by the Institutional Ethics Review Board of the Asan Medical Center (2013-0673), and all patients provided informed written consent before participating in the study. When next generation sequencing (NGS) was performed with punch biopsy specimens of lesional and perilesional facial skin tissue obtained from three patients with RM, GDA gene expression level increased 1,283.4, 3.7, and 26.4-fold in the facial lesions of 59-, 72-, and 59-year-old female patients with RM, respectively, than that in the non-lesional control areas (Table 1).

**Figure 1:**
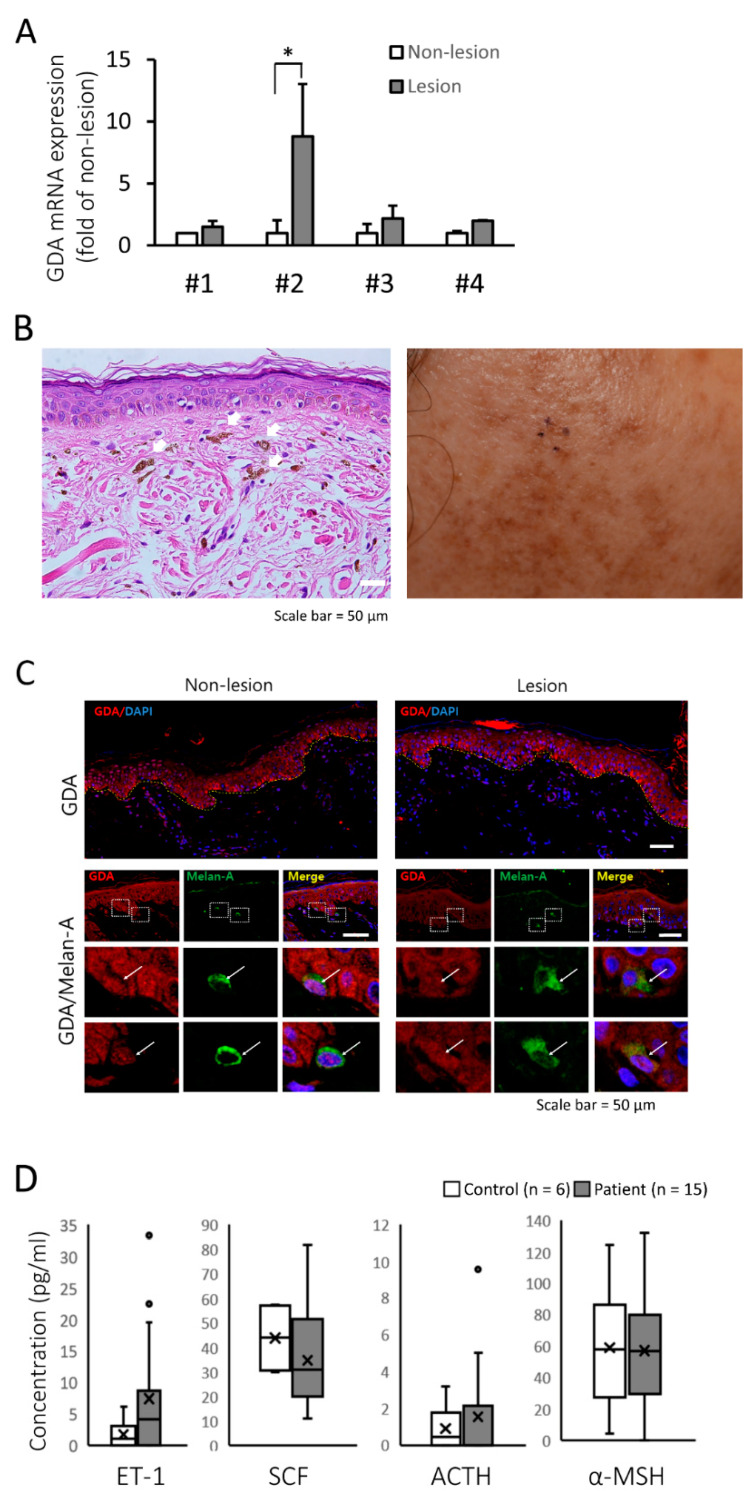
Guanine deaminase (GDA) expression in Riehl’s melanosis (RM) skin tissue. (**A**) Relative mRNA expression of GDA in lesional skin compared with that in non-lesional skin tissues from each patient with RM (n = 4). (**B**) Histopathologic (left panel, hematoxylin and eosin staining, original magnification 400×, dermal melanophages are indicated by white arrows) and clinical features (right panel) of a patient with RM. (**C**) Immunofluorescence and immunohistochemical staining for GDA in non-lesional and lesional skin from a patient with RM. The images demonstrated GDA (red) with 4′,6-diamidino-2-phenylindole (DAPI) (blue) as a nuclear counterstain (upper panel). Double immunofluorescence images for GDA (red), Melan-A (MART-1; melanocyte marker; green). Merged images with DAPI (blue) showed GDA and Melan-A double positive cells (white arrow; lower panel, scale bar = 50 μm). (**D**) Box plots displaying the minimum, the maximum, the median, and the first and third quartiles for serum endothelin-1 (ET-1), stem cell factor (SCF), adrenocorticotropic hormone (ACTH), and alpha-melanocyte-stimulating hormone (α-MSH) levels in patients with RM (n = 15) and age-matched healthy volunteers (n = 6). * *p* < 0.05.

We also performed quantitative PCR (qRT-PCR) in samples from four RM lesions and perilesional controls. The clinical information on the patients who participated in NGS and qRT-PCR analyses are shown in Supplementary Materials Table S1. GDA mRNA expression was higher in hyperpigmented lesions than in the controls (Figure 1A). Hematoxylin and eosin (H&E) staining of the hyperpigmented lesions showed epidermal hyperpigmentation with dermal melanophages (Figure 1B). Immunofluorescence staining showed that GDA was more strongly expressed in the epidermal KCs of RM lesions than in those of perilesional controls (Figure 1C). These results suggested that GDA expression in KCs is associated with skin hyperpigmentation. However, serum ET-1, SCF, ACTH, and α-MSH levels from 15 patients with RM were not significantly different from those in healthy volunteers (n = 6, Figure 1D), although serum ET-1 level was found to be higher than that in volunteers

### 2.2. GDA is Highly Expressed in Cultured Human KCs and Upregulated when Treated with an Inflammatory Cytokine Mixture

**Figure 2:**
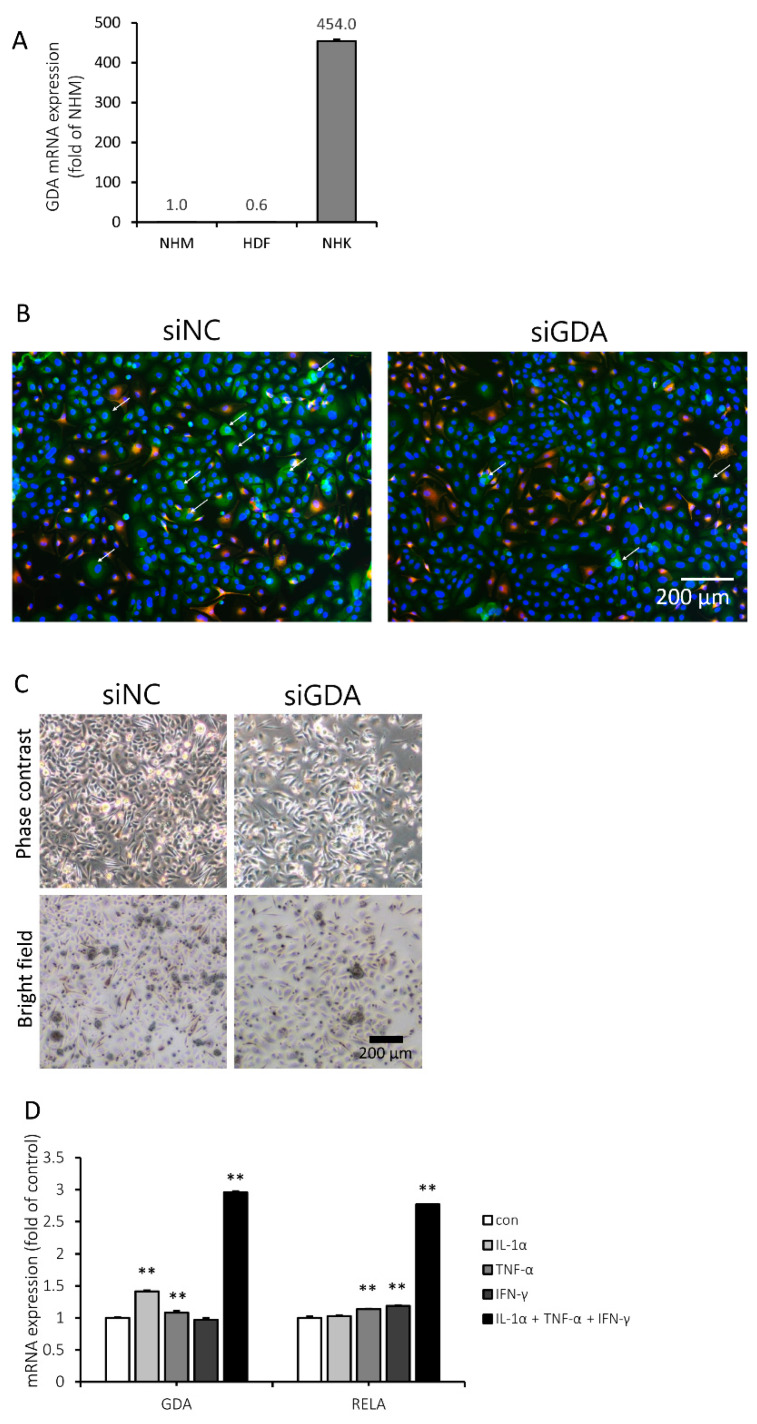
Guanine deaminase (GDA) was mainly expressed in human keratinocytes (KCs) and upregulated when treated with a mixture of inflammatory cytokines. (**A**) The relative GDA mRNA expression levels in normal human melanocytes (NHMs), human dermal fibroblasts (HDFs), and normal human keratinocytes (NHKs) were calculated after normalization to the RPLP0 gene. (**B**) NHMs cocultured with NHKs were transfected with negative control siRNA (siNC) or GDA (siGDA) for 3 d. To determine whether GDA expression level was decreased and to identify the GDA-expressing cells, the cells were double-stained with GDA (green) and Melan-A (red) and counterstained with 4′,6-diamidino-2-phenylindole (DAPI) (blue). (**C**) Pigmentary changes in cocultured cells by siGDA were demonstrated in bright-field and phase contrast images. (**D**) NHKs were stimulated with 4 ng/mL interferon gamma (IFN-γ), 20 ng/mL tumor necrosis factor alpha (TNF-α), and 20 ng/mL interleukin 1 alpha (IL-1α) for 24 h, and the GDA and RELA mRNA expression levels were the highest when treated with a mixture of the three cytokines. ** *p* < 0.01.

Next, we attempted to compare GDA expression in primary human KCs, fibroblasts, and melanocytes. To investigate whether GDA is constitutively expressed in cultured skin cells, we performed qRT-PCR. GDA mRNA expression in normal human KCs (NHKs) was nearly 100-fold higher than that in NHMs and human dermal fibroblasts (HDFs, Figure 2A). Thus, we focused on the role of GDA expression in KCs interacting with melanocytes to develop pigmentation.

When GDA was visualized by immunofluorescence staining in KC and melanocyte coculture, it was predominantly localized in the cytosol of KCs. Transfecting the coculture with a small interfering RNA for GDA (siGDA) suppressed the GDA signal in KCs (Figure 2B). In addition, melanosomes were far less abundant in siGDA-transfected cocultured cells than in cells transfected with negative control siRNA (siNC) (Figure 2C).

To determine whether GDA expression can be associated with inflammatory stimuli, human KCs were treated with various inflammatory cytokines, and the GDA mRNA expression level was evaluated by qRT-PCR. The expression level of RELA, a member of the nuclear factor-kappa B (NF-κB) family known to be involved in the inflammatory response [33,34,35], was also investigated. The GDA mRNA expression level was elevated with interleukin 1 alpha (IL-1α) or tumor necrosis factor alpha (TNF-α), while TNF-α or interferon gamma (IFN-γ) increased RELA mRNA expression. Both GDA and RELA mRNA expression levels were increased the most after treatment with a mixture of IL-1α, TNF-α, and IFN-γ (Figure 2D).

### 2.3. Representative Melanogenic Stimuli, UVB Irradiation and SCF/ET-1, are Associated with Increased Expression Level of GDA

**Figure 3:**
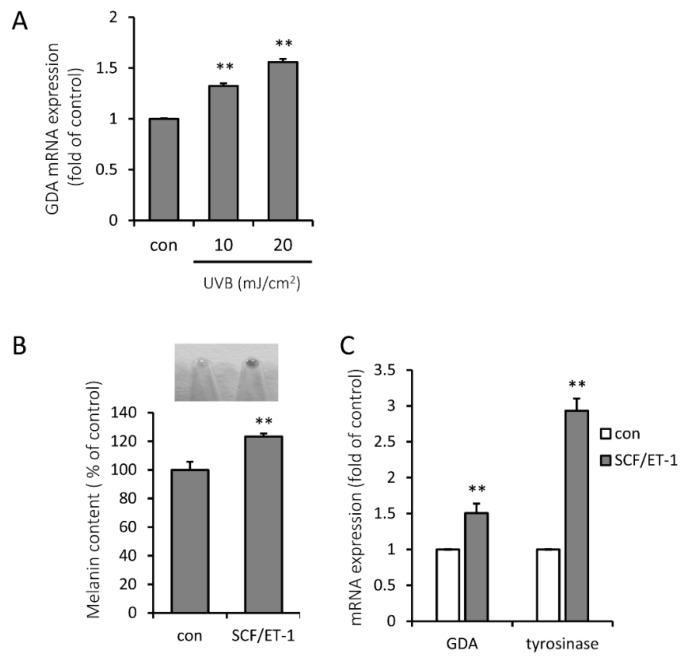
UVB irradiation and stem cell factor (SCF)/endothelin-1 (ET-1) upregulates guanine deaminase (GDA) expression. (**A**) GDA mRNA expression was increased in UVB-exposed normal human keratinocytes (NHKs) in a dose-dependent manner at 24 h. (**B**) Melanin content was significantly increased in the coculture of NHKs and normal human melanocytes (NHMs) when stimulated by 10 ng/mL SCF and 0.1 nM ET-1 for 5 d. (**C**) GDA and tyrosinase mRNA expression levels were increased by treatment with 10 ng/mL SCF and 0.1 nM ET-1 for 5 d in a coculture of NHKs and NHMs. ** *p* < 0.01.

KCs are the well-known neighboring cells to melanocytes, which secrete melanogenic mediators to melanocytes in the epidermis [1,2,3]. To investigate whether GDA can be induced by UVB irradiation in KCs, which is the most important physiologic triggering factor for hyperpigmentation, we measured GDA expression after exposure to low-dose UVB. Exposure of KCs to UVB led to an increase in GDA mRNA expression in a dose-dependent manner (Figure 3A).

UVB irradiation is known to increase melanogenesis by upregulating SCF and ET-1, melanogenic growth factors in KCs [3]. When we stimulated melanogenesis in a coculture of NHMs and human KCs by melanogenic growth factors from KCs, the combination of 10 ng/mL SCF and 0.1 nM ET-1 worked as the most consistent stimulator (Figure 3B). GDA and tyrosinase mRNA levels were significantly increased in the SCF/ET-1-costimulated coculture (Figure 3C).

### 2.4. siGDA in KCs Downregulates Melanogenesis While GDA Overexpression Promotes Melanogenesis in the Coculture

**Figure 4:**
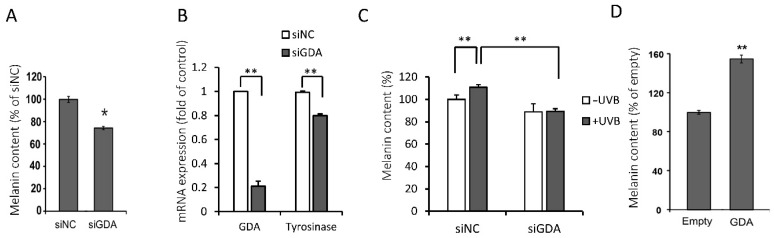
The use of small interfering RNA for guanine deaminase (siGDA) in keratinocytes (KCs) downregulates melanogenesis while guanine deaminase (GDA) overexpression promotes melanogenesis in the coculture. (**A**) Melanin content and (**B**) GDA and tyrosinase mRNA expression levels were decreased by siGDA in the coculture of normal human melanocytes (NHMs) and normal human keratinocytes (NHKs) for 5 d. (**C**) While UVB increased melanin content in the negative control siRNA (siNC)-transfected coculture of NHMs and NHKs, siGDA effectively reversed this accelerated melanin accumulation by UVB on day 5. (**D**) Melanin content was increased by a lentiviral-overexpressed GDA gene in the coculture of NHMs and HaCaT cells on day 5. * *p* < 0.05, ** *p* < 0.01.

To examine the association between GDA and melanogenesis, we silenced GDA using siGDA in the coculture, which significantly reduced the melanin content and tyrosinase mRNA expression on day 5 (Figure 4A,B).

Since we found that GDA mRNA level is increased under the UVB melanogenesis-stimulatory conditions in KCs, we sought to elucidate whether suppression of GDA expression also decreases the melanin content in the UVB-stimulated coculture. Melanin content was even lower in the UVB-stimulated coculture of KCs and melanocytes after GDA suppression in KCs by siGDA than in the coculture without UVB stimulation, suggesting the regulating role of GDA in UVB-mediated melanogenesis (Figure 4C).

Next, we attempted to induce GDA overexpression using an adenovirus vector. While NHMs and NHKs could not be infected with GDA–adenovirus, HaCaT cells were successfully infected. Increased GDA expression in HaCaT cells promoted melanogenesis in a 1:1 coculture of NHMs and GDA-overexpressed HaCaT cells (Figure 4D).

### 2.5. KC GDA Expression is Involved in the Melanogenic Property of UV Treated KC-Conditioned Media

**Figure 5:**
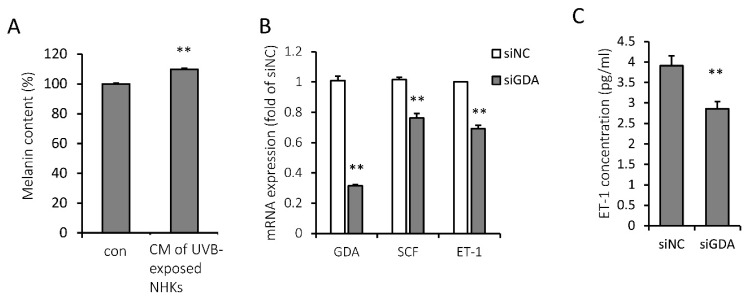
Keratinocyte (KC) guanine deaminase (GDA) expression is involved in the melanogenic property of UV-exposed normal human keratinocyte (NHK)-conditioned media. (**A**) Melanin content was increased when normal human melanocytes (NHMs) were treated with UVB-exposed NHK-conditioned media (CM) for 5 d. (**B**) Stem cell factor (SCF) and endothelin-1 (ET-1) mRNA expression levels in small interfering RNA for guanine deaminase (siGDA)-transfected NHKs were significantly lower than those in negative control siRNA (siNC)-transfected NHKs at 48 h. (**C**) ET-1 concentration was reduced in the conditioned media of siGDA-transfected NHKs at 48 h. ** *p* < 0.01.

To investigate how KC GDA regulates melanocyte function, we treated melanocytes with UVB-treated KC-conditioned media, which increased the melanin content (Figure 5A). ET-1 and SCF are known to be secreted from KCs after UVB irradiation and subsequently act on melanocytes via c-KIT or endothelin receptor type B to accentuate melanogenesis [3]. ET-1 and SCF mRNA expression levels were significantly lower in siGDA-transfected NHKs than in siNC-transfected NHKs, suggesting GDA involvement in the regulation of ET-1 and SCF production from KCs (Figure 5B). In addition, ET-1 concentration was significantly lower in the media of siGDA-transfected NHKs than those of siNC-transfected NHKs on day 2 (Figure 5C).

## 3. Discussion

Skin pigmentation is regulated by many factors and is complicated by the interconnections between melanocytes, KCs, and fibroblasts. Interactions between KCs and melanocytes under UVB exposure are reported [1,2,3,4,5,6,11]. The interactions are via direct cell contact and/or by secretion of various paracrine factors. Among the constituent cells present in skin, KCs secrete many mitogenic or melanogenic factors recognized by corresponding receptors on melanocytes. These KC-derived cytokines or growth factors include ET-1, SCF, α-MSH, ACTH, basic fibroblast growth factor, prostaglandins, and nitric oxide [1,2,3,11,36,37,38,39,40,41,42,43]. Particularly, ET-1 and SCF are the two major factors regulating the biology of human epidermal melanocytes. UV radiation exposure stimulates pigmentation of human skin, and epidermal melanocytes produce melanosomes that are transferred to the surrounding KCs in response to paracrine factors released under UV stimulation [11,38,39,40,41,42,43]. UVB stimulates ET-1 receptor expression in melanocytes via the sequential activation of the p38/Mitogen- and stress-activated kinase (MSK) 1/cyclic adenosine monophosphate response element-binding protein (CREB) pathway [38]. SCF interacts with ET-1 while inducing melanogenesis in response to UV irradiation via ET-1 receptor upregulation in melanocytes [40]. Upon UV stimulation, membrane-bound SCF level and secretory ET-1 production are increased in KCs [36,40].

In our previous study using DNA microarray from melasma lesions, GDA was shown to be one of the most upregulated genes, which was validated by qRT-PCR analysis [17]. KCs were the most abundant type of cells obtained from a skin biopsy using a 2 mm punch, and high GDA expression in cultured KCs suggested that KCs are the source of GDA in hyperpigmented lesions. In this study, we have shown that GDA expression level is also higher in RM lesions than in non-lesional controls. RNA microarray analysis showed that GDA expression level was increased in RM lesions, but its expression in sporadically-occurring congenital hyperpigmentary lesions of café au lait macules was lower than that in perilesions in our preliminary study (data not shown). RM is a peculiar severe form of post-inflammatory hyperpigmentation (PIH) on the face and neck of dark-skinned adults, which is triggered by skin irritation or contact dermatitis and aggravated by UV exposure [44].

Based on the above results, we hypothesize that epidermal KC GDA expression promotes melanogenesis. Our present study showed that GDA in epidermal KCs may promote pigmentation and play a role in UV-induced melanogenesis by interacting with KC-derived melanogenic growth factors ET-1 and SCF, suggesting its role in UV-aggravated skin hyperpigmented disorders. These results suggest that inhibiting keratinocyte GDA expression can be a novel approach for treating skin hyperpigmentary disorders.

## 4. Materials and Methods

### 4.1. Patients

We included patients who were clinically diagnosed with RM in the Dermatology Department of the Asan Medical Center (Seoul, Korea). The patients were excluded if they had other possible causes of hyperpigmentation such as Addison’s disease, hyperthyroidism, and hemochromatosis. We also excluded patients with hyperpigmentation in sites other than the face and neck. Clinical features such as age, sex, location of the lesion, Fitzpatrick skin type, morphology of the lesion (spotty, reticulated, diffuse, presence of erythema of hypopigmentation), and color of the lesion (bright/average, dark/very dark) were identified.

### 4.2. Materials

NHMs, NHKs, and HDFs were obtained from Invitrogen (Carlsbad, CA, USA). Anti-GDA and anti-Melan-A antibodies were obtained from GeneTex (Irvine, CA, USA) and Cell Marque (Rocklin, CA, USA), respectively. SCF, ET-1, IL-1α, and IFN-γ were purchased from R&D systems (Minneapolis, MN, USA), and TNF-α was purchased from BioSource (Camarillo, CA, USA). siGDA, siNC and all primers (GDA, RELA, tyrosinase, SCF, ET-1, RPLP0) were purchased from Bioneer (Daejeon, Korea).

### 4.3. Next Generation Sequencing (NGS)

We performed punch biopsies of lesional skin and normal adjacent skin on 3 RM patients (one biopsy specimen for lesional and non-lesional control area, respectively, per person). Total RNA was isolated from tissue using the Trizol based method. One microgram of total RNA was processed for preparing the mRNA sequencing library using the TruSeq stranded mRNA sample preparation kit (Illumina, San Diego, CA, USA) according to the manufacturer’s instructions. The first step involves purifying the poly-A containing mRNA molecules using poly-T oligo attached magnetic beads. Following purification, the mRNA is fragmented into small pieces using divalent cations under elevated temperature. The cleaved RNA fragments are copied into first strand cDNA using reverse transcriptase and random primers. Strand specificity is achieved by replacing dTTP with dUTP in the second strand marking mix (SMM), followed by second strand cDNA synthesis using DNA polymerase I and RNase H. These cDNA fragments then have the addition of a single ’A’ base and subsequent ligation of the adapter. The products are then purified and enriched with PCR to create the final cDNA library. Finally, quality and band size of libraries were assessed using an Agilent 2100 bioanalyzer (Agilent, Santa Clara, CA, USA). Libraries were quantified by qPCR using the CFX96 Real Time System (Biorad, Hercules, CA, USA). After normalization, sequencing of the prepared library was conducted on the Nextseq system (Illumina) with 75 bp paired-end reads. The result was aligned using the reference human genome (hg19). Three independent analyses were performed per biopsy specimen.

### 4.4. Expression Analysis by qRT-PCR

Total RNA was isolated from skin biopsies (one biopsy specimen for lesional and non-lesional control area, respectively, per person) or cells using the RNeasy Mini kit (Qiagen; Valencia, CA, USA). Then, 1 μg RNA was reverse-transcribed using the RevertAid First Strand cDNA Synthesis Kit (Invitrogen). qRT-PCR was performed using the LightCycler^®^ 480II machine coupled with SYBR Green (Roche Applied Science; Indianapolis, IN, USA). For qRT-PCR, the initial denaturation was performed at 95 °C for 5 min, followed by amplification at 95 °C for 10 s, 60 °C for 10 s, and 72 °C for 10 s for 45 cycles. Relative gene expression levels were calculated after normalization to the RPLP0 gene using the ΔΔCt method. Three independent analyses were performed per biopsy specimen. Three independent cellular experiments were performed in triplicate.

Primers for RPLP0 were used for loading control amplifications. Specific primer sets used for each gene are shown in Table 2.

### 4.5. Immunofluorescence Staining and Serum Analysis

For immunofluorescence staining, the samples obtained from RM lesional skin and adjacent perilesional skin were fixed in 10% neutral buffered formalin and embedded in paraffin. The specimens were cut into 4 μm-thick sections, and serial sections were deparaffinized and rehydrated. For antigen retrieval, the sections were heated in antigen unmasking solution (Vector Laboratories; Burlingame, CA, USA) using a pressure cooker (Biocare Medical, Pacheco, CA, USA) at 120.5 °C for 30 s and 90 °C for 10 s. Then, they were immunofluorescently stained with the primary antibodies for GDA (1:200) and Melan-A (1:50) at 4 °C for 8 h. We used a FITC-conjugated anti-mouse (1:500) secondary antibody to detect Melan-A and anti-rabbit Alexa Fluor 546 (1:500) to detect GDA at 4 °C for 30 min. Images were acquired using a Zeiss LSM 780 laser scanning confocal microscope (Leica; Wetzlar, Germany).

Blood samples were obtained from 15 patients with RM (females; age: 51.3 ± 10.0 years; body mass index (BMI): 23.6 ± 2.7) and 6 healthy volunteers (females; age: 39.7 ± 3.6 years; BMI: 21.3 ± 2.9). The serum was separated by centrifugation at 4000× *g* for 10 min at 4 °C and stored at −70 °C. ET-1 and SCF levels in the serum were measured using the Human Magnetic Luminex Screening Assay kit (R&D systems; Minneapolis, MN, USA) according to the manufacturer’s instructions. ACTH and α-MSH serum levels were measured using the MILLIPLEX MAP Human Pituitary Magnetic Bead Panel (Merck Millipore; Burlington, MA, USA) and the MILLIPLEX MAP Human Neuropeptide Magnetic Bead Panel (Merck Millipore; Burlington, MA, USA), respectively, according to the manufacturer’s instructions.

### 4.6. Cell Culture and Melanin Content Assay

NHMs were maintained in Medium 254 (Invitrogen) containing human melanocyte growth supplement (Invitrogen). NHKs were cultured in KC growth medium (EpiLife; Invitrogen) supplemented with human KC growth supplement (Invitrogen). HDFs and HaCaTs were cultured in Dulbecco′s modified Eagle′s medium (DMEM) supplemented with 10% fetal bovine serum (FBS). All cells were maintained at 37 °C in a humidified atmosphere with 5% CO_2_. For coculture of NHMs and NHKs, 6 × 10^4^ NHM cells were seeded in each well of a 6-well plate. The next day, 3 × 10^5^ NHK cells were added to each well for the coculture. NHMs and NHKs were cocultured at a 5:1 seeding ratio in KC growth medium.

Cells were dissolved in 550 μL 1 N NaOH at 100 °C for 30 min and centrifuged at 13,000 rpm for 5 min. The optical density of the supernatant was measured at 405 nm using a microplate reader (Molecular Devices, Sunnyvale, CA, USA).

### 4.7. Knockdown and Ectopic Expression of GDA

siGDA and siNC were transfected into cells using the Lipofectamine^®^ RNAi MAX reagent (Invitrogen) according to the manufacturer’s instructions. The siGDA sequence is AGUUGUCAGGAGAACACUA. The detailed knockdown protocol is shown in Table 3.

For ectopic expression of GDA, human GDA cDNA was cloned into lentiviral vectors (pcDH-CMV-EF1-puro plasmids (System Biosciences, Mountain View, CA, USA)) and lentiviruses containing either empty vector (Empty) or human GDA (GDA) were produced and introduced into HaCaT cells. After puromycin selection and confirmation of GDA overexpression by qRT-PCR, HaCaT cells overexpressing GDA or infected with empty virus were cocultured with NHMs for 5 d and melanin content was measured as described above.

### 4.8. UVB Radiation and Preparation of KC-Conditioned Media

The cells were exposed twice to a narrow band-UVB lamp (Dermalight Psoracomb UV-B-311 nm-narrowband; National Biological Corp.; Beachwood, OH, USA). The cells were washed and resuspended in phosphate-buffered saline (PBS) prior to UVB radiation exposure. Non-exposed control samples were maintained in the dark under the same conditions. Following UVB radiation exposure, the cells were grown in fresh medium.

NHK cells (6 × 10^5^) were seeded into a 60 mm dish, cultured for 24 h, washed with PBS, and treated UVB or siGDA. After treatment, the cells were incubated in KC growth medium for 48 h; the conditioned media was harvested, centrifuged, and stored at −20 °C.

ET-1 levels in the conditioned media were measured using an ELISA kit (R&D Systems; Minneapolis, MN, USA) according to the manufacturer’s instructions.

### 4.9. Statistical Analysis

The experimental data were expressed as means and standard deviations for three independent experiments performed in triplicate. Differences between results were assessed using the Kruskal–Wallis test in Figure 1 and Student’s t-test in Figure 2, Figure 3, Figure 4 and Figure 5. *p*-Values < 0.05 were considered significant.

## Figures and Tables

**Table 1:** The results of next generation sequencing for three patients with Riehl’s melanosis.

Patient	Sex	Age	Normalized GDA Gene Expression in Non-Lesion	Normalized GDA Gene Expression in Lesion	Fold Change	*p*-Value
1	Female	59	0.001	1.283	1283.4	<0.001
2	Female	72	0.432	1.616	3.7	0.109
3	Female	59	0.325	8.577	26.4	0.002

**Table 2:** Specific primer sets for each gene.

Name	Forward (5′ to 3′)	Reverse (5′ to 3′)
GDA	GCAACAATTCACACTGACTCATC	GTGTCACTATGGGCTTCACTC
RELA	ATGTGGAGATCATTGAGCAGC	CCTGGTCCTGTGTAGCCATT
SCF	AATCCTCTCGTCAAAACTGAAGG	CCATCTCGCTTATCCAACAATGA
ET-1	AAGGCAACAGACCGTGAAAAT	CGACCTGGTTTGTCTTAGGTG
RPLP0	GGCGACCTGGAAGTCCAACT	CCATCAGCACCACAGCCTTC

**Table 3:** The protocol for knockdown of guanine deaminase.

Day	1	2	3	4	5	6	7	8
Protocol	MCs seeding	KCs seeding	siRNA transfection for 24 h	Media change		Media change		Harvest

## Supplementary Materials

The following are available online at https://www.mdpi.com/1420-3049/25/11/2637/s1. Table S1: Clinical features of patients with Riehl’s melanosis who were included in next generation sequencing and quantitative PCR, Figure S1: The standard curve with synthetic melanin, Figure S2: Phase contrast images of keratinocyte-melanocyte coculture for 5 days.
