# Supplementary material for: Guanine Deaminase in Human Epidermal Keratinocytes Contributes to Skin Pigmentation
Source: Molecules. 2020 Jun 5;25(11):2637. doi: 10.3390/molecules25112637 (PMC7321356; doi:10.3390/molecules25112637)
Supplement: Supplementary file 1 [file molecules-25-02637-s001.pdf]

Supplementary Materials

Table S1. Clinical features of patients with Riehl's melanosis who were included in next generation sequencing and quantitative PCR

Next generation sequencing					
Patient number	Age	Sex	Location	Fitzpatrick skin type	Description of skin lesions
1	59	F	neck	IV	reticulated dark brown patches
2	72	F	cheek	IV	diffuse brownish patches
3	59	F	cheek	IV	diffuse dark brown patches
Quantitative PCR					
2	72	F	cheek	IV	diffuse brownish patches
4	80	M	forehead	IV	reticulated brownish patches
5	45	F	cheek	IV	diffuse dark brown patches
6	67	M	neck	IV	reticulated dark brown patches

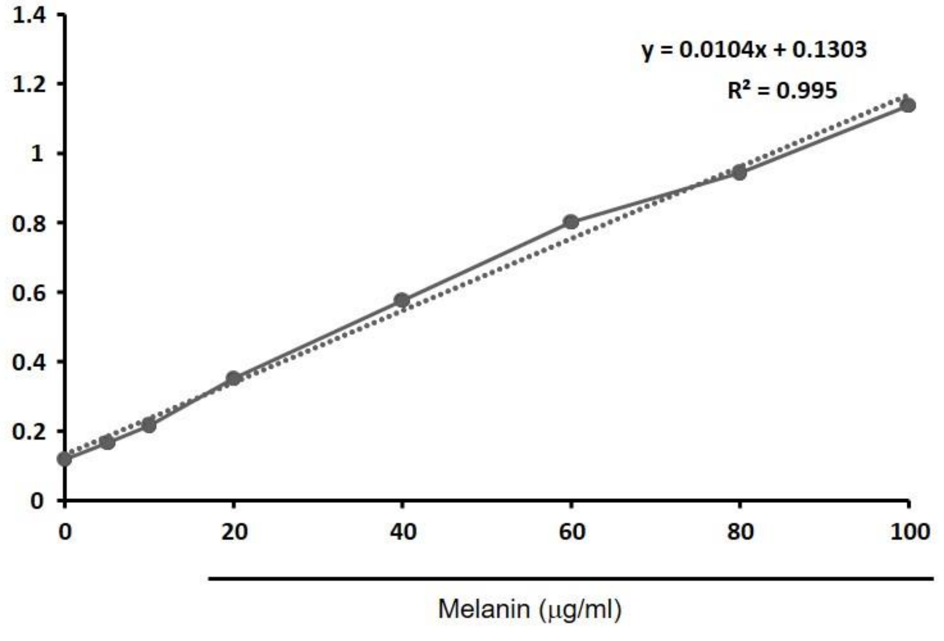

Figure S1. The standard curve with synthetic melanin

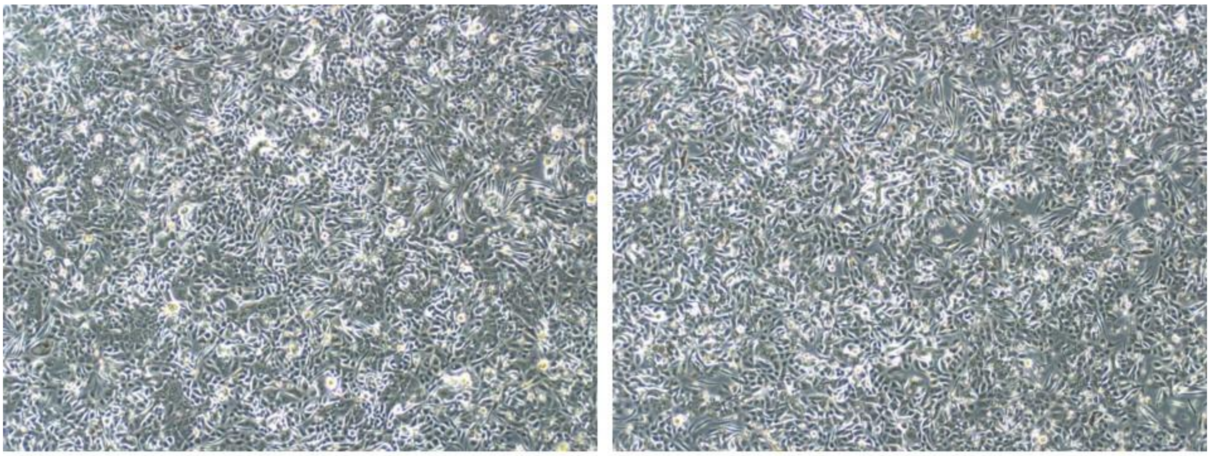

siNC siGDA

Figure S2. Phase contrast images of keratinocyte-melanocyte coculture for 5 days
